# Case of Cancer of the Breast Terminating Fatally by Trismus

**Published:** 1818-11

**Authors:** 


*For the London Medical and Physical Journal.*

### *Case of Cancer of the Breast terminating fatally by Trismus.*

By P. T.

IN the beginning of the year 1818, I was desired to visit Mrs. P. a healthy corpulent lady, in the 47th year of her age; she had borne two children, neither of which she had suckled; neither had she any milk. She was not married till the year 1813, and her youngest child is about three years old; since whose birth Mrs. P. had been regular as to menstruation, and enjoyed rather an extraordinary share of good health and spirits, excepting some fears which occasionally crossed her mind respecting the complaint in her breast.

I found nearly the whole glandular substance in a truly scirrhous state, ulcerated on a considerable portion of its external surface; the nipple destroyed; the discharge not profuse, nor particularly offensive; no tumor in the axilla, but a line of scirrhous hardness proceeding towards the clavicle, underneath which it evidently proceeded into the thorax, to which some difficulty of breathing was attributed. A dreadful, dry, and convulsive cough attacked her several times in the day and night, which could not be restrained by bleeding and large doses of opium.

I considered the case a hopeless one, and had recourse to such means as appeared to me best calculated to palliate her sufferings.

With this view, I directed the ulcerated surface of the breast to be besprinkled with ferri carbonas, covering it with dry lint, and the whole breast to be kept constantly cool and moist by the repeated application of soft cloths moistened with a weak solution of plumbi superacetas. The bowels were kept rather loose by a solution of magnesiæ sulphas in infusion of rose-leaves, given in small doses three or four times a-day. A sparing, chiefly vegetable, diet was enjoined; wines and fermented liquors altogether prohibited; and at bed-time a sound dose of opium. From fifty to sixty drops of tinctura opii was generally had recourse to, with the privilege of repeating it at three or four o'clock in the morning, if the cough, which was at this period the most afflicting symptom, should require it.

Under the above treatment the disease of the breast made no advance, but, on the contrary, it was entirely free from pain, and was manifestly diminished in size and hardness; but the scirrhous line was still traceable into the thorax. Her health was tolerably good, and the cough somewhat abated, or rather occurred less frequently; so that she could not easily have been persuaded that she should not perfectly recover. But this delusion was of short duration: at the beginning of March her cough was much aggravated, both in force and frequency, which she attributed to having, as she supposed, taken cold, in which opinion she was confirmed by a particular stiffness of the muscles of the lower jaw. This latter symptom increased very rapidly, with an incapability of swallowing either solids or fluids; consequently,

I was sent for in haste. The distance from Leicester being seven miles, it was several hours before I reached my patient; when, on the first sight of her sister, who was anxiously expecting my arrival, I became impressed with a conviction that something out of the common course of the disease had occurred; and so, indeed, I found it. I should here observe, that, in the early part of my attendance on the case, I consulted Mr. Abernethy, who recommended a steady perseverance in the plan already begun. Accordingly no change of treatment, either internal or external, was ever made, until the commencement of the tragical scene, which I am about to relate.

On entering my patient's room, I found her in great distress; almost constantly coughing with violence; unable to open her mouth, excepting by the forcible use of her hands, by which she could depress the jaw to a small degree; but, as she observed, it always snapped-to again on the hands being taken from it. She was sensible of the increasing violence of all her sufferings, to which was now added an indescribable intensity of thirst, with a sense of burning heat in the fauces, œsophagus, down into the stomach. The total impossibility of reaching the fauces with any kind of fluid, so earnestly desired by her, added, in no small degree, to her afflictions. Her efforts to swallow were made as resolutely as possible, but they were always frustrated; for, the moment the fluid was drawn within the mouth, long before it reached the fauces, she was seized with convulsions, in all respects resembling those in the hydrophobia, though she did not express any abhorrence of fluids, but was rather pleased with the application of cold water to the surface of the skin. I bled her freely, applied leeches, directed the warm bath, and an opiate enema at bed-time; ung. hydrarg. fort. to the neck and jaws.

The next morning all the symptoms were aggravated, with tetanous rigidity of the abdominal muscles superadded. Under these circumstances, I requested another opinion: accordingly, Dr. William Arnold accompanied me. We examined the breast attentively, but the appearance was as favourable as usual; neither was the scirrhous line proceeding into the thorax either more large or more tender; and, what is somewhat remarkable, no part of the breast had ever, from the beginning of the disease to the present time, given any uneasiness, not even one lancinating pain. The applications to the breast were repeated; and, as all medicine and food were absolutely precluded by the mouth, we were compelled to employ external applications and enemas. The warm bath; equal weights of extractum opii mol. and ung. hydrar.

fort. to be mixed, and assiduously rubbed on the neck and jaws three times a-day; in the intermediate times keeping the parts cold by the perpetual application of cold water. An enema of broth and milk to be administered three times a-day, and to that at bed-time the addition of two drachms of tinctura opii.

The morning following, in every respect worse; much fatigued by the administration of clysters; had passed two stools; thirst intense; skin hot; pulse feeble, and rather slow; cough almost incessant, and convulsive; countenance dejected, pitiable. No hope remained; the last effort made to lessen human misery (for she now declined any more exhibitions of clysters, the last having so exhausted her that it was expected she would not have survived it,) was the external application of tincture of opium to the surfaces and the rigid muscles. It was incessantly applied, and in ten hours not less than a pint of the tincture was used. At first she felt relief and much comfort from it, and conceived that in it she had found a permanent friend. She slept, at intervals, as long as fifteen minutes at a time, but awoke always with cough and convulsions. The convulsive struggles increased in frequency and strength; and, immediately after a remarkably strong one, she was exhausted, and died on the fourth day after the commencement of trismus, about five o'clock in the morning.

Examination *post mortem* was not permitted.

Having already trespassed beyond the limits allowable to an individual case, I shall reserve such observations on the disease commonly called Cancer as I hope will illustrate its real nature, and afford it an appropriate appellation.

*Leicester; Oct.* 10, 1818.

---

*For the London Medical and Physical Journal.*

